# Manual versus forceps postplacental IUD insertion after vaginal delivery: A randomized clinical trial

**DOI:** 10.1002/ijgo.70355

**Published:** 2025-07-07

**Authors:** Thuany Bento Herculano, Andressa Aparecida Luciano Batista, Cássia Raquel Teatin Juliato, Patrícia Moretti Rehder, Fernanda Surita

**Affiliations:** ^1^ Department of Obstetrics and Gynecology, School of Medical Science University of Campinas São Paulo Brazil

**Keywords:** contraception, immediate postplacental insertion, intrauterine device, postpartum period, vaginal delivery

## Abstract

**Objectives:**

This study compares the expulsion rate of the postplacental copper intrauterine device (IUD) based on the insertion technique: manual versus ring forceps. Pain, perforation, infection, and increased bleeding were assessed as secondary outcomes.

**Method:**

The study consisted of a randomized clinical trial involving 210 women (with 105 in each group) admitted for vaginal delivery at a teaching hospital who wanted to use an IUD and had no contraindications to use an IUD. Patient recruitment occurred between December 2021 and June 2024. Pain was evaluated using the visual analog scale immediately after insertion. Descriptive statistics and bivariate analysis were performed. The Cochran–Armitage test was used to assess the temporal trend of expulsion. Participants were followed up between 6 and 12 weeks postpartum.

**Results:**

The complete IUD expulsion rate was 20.5%, with no significant difference between forceps and manual insertion (25% vs. 16.7%, *P* = 0.168). Expulsions were easily perceived by the women and occurred early. Notably, in this teaching hospital setting, the turnover of new medical residents (*P* = 0.057) performing insertions did not affect the expulsion rates throughout the year. Manual insertion was associated with greater discomfort compared to forceps (*P* = 0.001), even with epidural anesthesia (*P* = 0.002). There were no cases of uterine perforation or infection.

**Conclusion:**

The postplacental IUD expulsion rate was lower with manual insertion, although not statistically significant. Insertion discomfort was lower in the forceps group, regardless of analgesia. Postplacental IUD insertion was well tolerated, safe, and easily reproducible by junior doctors.

**Trial registration:**

This study was approved by the Ethics and Research Commission of the University of Campinas (UNICAMP) and the Brazilian Registry of Clinical Trials (REBEC): https://ensaiosclinicos.gov.br/rg/RBR‐4j62jv6, (number RBR‐4j62jv6).

## 1 INTRODUCTION

Despite declining fertility rates, inequalities in access to reproductive planning limit contraception and legal abortion services for women with lower income and education, especially in low‐ and middle‐income countries.^1^ These inequities particularly affect women who are socially more vulnerable to unplanned pregnancies: adolescents, multiparous women, psychoactive substance users, and those in remote areas.^2^, ^3^, ^4^, ^5^

Long‐acting reversible contraceptives (LARCs), such as intrauterine devices (IUDs), are ideal in such cases due to their efficacy, duration, safety, and minimal need for follow‐up visits to healthcare services.^2^, ^3^, ^4^ Hospitalization for childbirth care is often the only opportunity for some women to access reproductive planning.^5^

Long‐acting reversible contraceptive access is also limited by a shortage of trained providers, mainly concentrated in major urban centers and university institutions. This is especially true for immediate postpartum insertion, which is technically distinct and still infrequently taught.^6^

After a vaginal delivery, the IUD can be inserted either manually or with a long, ring‐tipped forceps, such as the modified Kelly forceps, which is specifically designed for this purpose and lacks a ratchet to avoid damaging the copper on the IUD. A long inserter developed for this technique is also available; however, it is not widely accessible and has been associated with even higher expulsion rates compared to other techniques. This is likely due to the longer visible string during the early postpartum period, which might lead to inadvertent traction.^7^

There is a lack of randomized clinical trials comparing IUD insertion techniques immediately after vaginal delivery. The primary goal of the various proposed methods is to minimize the main limitation of postplacental insertion—a higher expulsion rate compared to cesarean or interval placement^8^, ^9^, ^10^—without increasing other potential complications, such as perforation or intrauterine infection. Therefore, this randomized clinical trial was conducted to compare two postplacental TCu380A IUD insertion techniques: manual versus forceps.

## 2 METHOD/DESIGN

### 2.1 Trial design

This was an open‐label, parallel‐group (1:1) randomized trial.^11^ This study was approved by the Ethics and Research Commission of the University of Campinas (UNICAMP) (CAAE: 50497321.4.0000.5404) and registered with the Brazilian Registry of Clinical Trials (REBEC): https://ensaiosclinicos.gov.br/rg/RBR‐4j62jv6, (number RBR‐4j62jv6). The trial followed CONSORT guidelines,^11^ and its protocol was previously published.^12^

### 2.2 Sample size

The calculation of the sample size was performed by comparing two groups, considering a 5% significance level and 80% power.^13^ Based on the results, a sample of *n* = 186 women (*n* = 93 with manual insertion and *n* = 93 with forceps insertion) was estimated to be representative for comparing expulsion rates between the two groups (19.08% vs. 37.50% expulsion, respectively, according to data available in the literature).^14^ Considering a 10% loss during follow‐up, 210 participants were included.

### 2.3 Participants

All women received prior counseling on the contraceptive methods available at the hospital during one of their prenatal consultations. They were also informed about the high efficacy of the copper IUD and its potential side effects.

Women aged between 18 and 43 years, with a single pregnancy beyond 37 weeks, hemoglobin ≥8.0 mg/dL, and who desired a copper IUD were invited to participate. Women with uterine malformations or fibroids distorting the uterine cavity, diagnosed chorioamnionitis or active sexually transmitted infections, or indications for elective or emergency cesarean section were excluded prior to randomization.

Eligible women signed an informed consent form and were randomized. Participants were withdrawn if they chose to leave the study or presented fever during labor, had ruptured membranes for more than 24 h, required manual placenta extraction, or experienced postpartum hemorrhage or uterine atony. Each participant received a number corresponding to her position on the randomization list and was identified by this number throughout the follow‐up.

### 2.4 Setting

The study was conducted at the Women's Hospital (CAISM) of UNICAMP, Brazil. This tertiary hospital is a reference center in the city of Campinas, São Paulo State, specializing in high‐risk pregnancies and serving as a center of excellence for training new gynecologists.

### 2.5 Randomization

The research team randomized the eligible women to one of the insertion techniques (manual or forceps) using a randomization sequence generated by software developed by a statistician. Block randomization was performed using fixed‐size blocks of 10 participants, ensuring an equal number of each insertion type within each block, distributed in a random order.

Allocation was concealed using opaque, sealed, and sequentially numbered envelopes. The envelope containing the randomization result was opened at the time of insertion (during placental expulsion). Healthcare professionals and the research team blinded participants to the insertion method. Randomization took place from December 1, 2021, to June 24, 2024.

### 2.6 Outcomes

The primary outcome was IUD expulsion reported by the patient within 12 weeks postpartum. Secondary outcomes included pain reported immediately after insertion (assessed using the visual analog scale [VAS]), occurrence of puerperal endometritis, and prolonged lochia lasting more than 6 weeks, all evaluated according to the insertion technique. Endometritis was clinically defined by fever, abdominal and pelvic pain, and foul‐smelling vaginal discharge.^15^

### 2.7 Data collection

Data were collected using a structured questionnaire administered by a research team member at three time points: (i) upon admission for delivery, gathering obstetric history and sociodemographic characteristics; (ii) within 1 h postpartum, recording delivery details (e.g., induction, time of membrane rupture, analgesia, instrumental delivery, and perineal laceration) and IUD insertion (at this stage, the VAS was applied); and (iii) at the postpartum follow‐up visit between 6 and 12 weeks, collecting information on IUD expulsion, participant satisfaction, increased bleeding, or other clinical complaints.

### 2.8 Procedures

Resident training in both postplacental IUD (ppIUD) insertion methods remained consistent throughout the patient recruitment period. Training consisted of a ppIUD insertion simulation at the beginning of the intern year, followed by bedside training with senior residents and attending physicians serving as instructors. Junior residents were the primary proceduralists. Insertions occurred in the delivery room within 10 min of placental expulsion.

**FIGURE 1:**
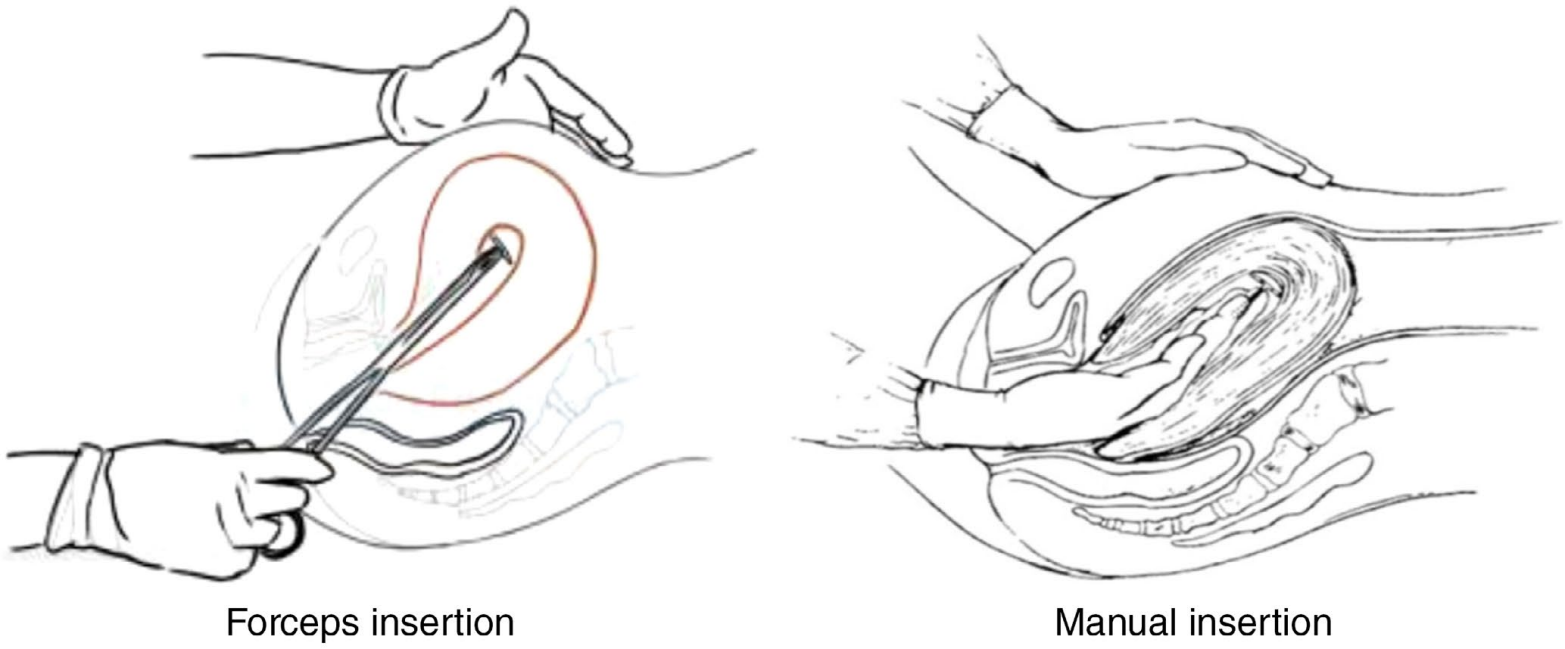
Ring forceps insertion and manual insertion.

Before the insertion, asepsis of the vulva and vagina was performed using an aqueous chlorhexidine solution. For insertions with forceps, the anterior lip of the cervix was grasped with a Collin forceps, and the IUD was gently guided to the uterine fundus while secured in a modified Kelly forceps. The Kelly forceps were suitable due to their 32‐cm length (sufficient to reach the uterine fundus), ring‐shaped tip, and lack of ratchet, allowing a firm grip without damaging the IUD. In manual insertions, the physician's non‐dominant hand remained at the uterine fundus while the dominant hand guided the IUD to the uterine fundus (Figure 1). A demonstration video of the insertion techniques is available in the study protocol.^12^

Patients were advised to visit the hospital's emergency department if they had complaints (e.g., pain, bleeding, or fever) or suspected IUD expulsion. The study site operates continuously, 24 h a day. A follow‐up visit was scheduled 6–12 weeks postpartum. Women who did not seek care or missed follow‐up were contacted by phone to assess complaints, reschedule, and inquire whether they had observed the expulsion of the device.

### 2.9 Statistical analysis

Coded data were anonymized and stored in an Excel database created for this purpose. The database is publicly available in UNICAMP's official data repository.^16^

To describe the sample profile, tables were created with absolute frequency values (*n*) and percentages (%), as well as descriptive statistics for numerical variables. Group comparisons used *χ* ^2^ or Fisher's exact test and the Mann–Whitney test. The Cochran–Armitage test was used to assess temporal trends. A 5% significance level was adopted. Statistical analyses were performed using the Statistical Analysis System (SAS), version 9.4.

## 3 RESULTS

Between December 2021 and June 2024, there were 1202 vaginal deliveries, and a total of 210 women were randomized for postplacental IUD insertion after vaginal delivery using either the manual technique (*n* = 105) or ring forceps (*n* = 105). The participants' mean age was 25.9 ± 6.2 years. Labor was spontaneous in 53.3% (*n* = 112) and induced in 46.7% (*n* = 98). Most participants had two or more pregnancies (59.5%) and did not wish to bear additional children (82.4%).

In the “Manual” group, 6.7% (*n* = 7) were withdrawn before insertion due to increased uterine bleeding (*n* = 5) or manual placental extraction (*n* = 2). In the “Forceps” group, 7.6% (*n* = 8) were withdrawn due to increased bleeding (*n* = 4), manual placental extraction (*n* = 2), or expressing the desire to leave the study (*n* = 2).

Of the 195 insertions (Manual = 98; Forceps = 97), follow‐up loss at 12 weeks was 2% (*n* = 2) in the Manual and 13.4% (*n* = 13) in the Forceps group.

**FIGURE 2:**
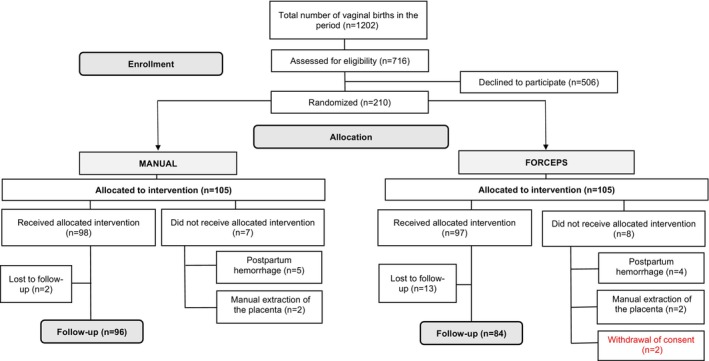
Flowchart (CONSORT diagram).

A participant flowchart is presented in Figure 2.

Baseline sociodemographic and obstetric characteristics were similar between the study groups (Table 1).

**TABLE 1:** Main sociodemographic and clinical characteristics of women randomized for postplacental TCu380A IUD placement after vaginal delivery, categorized by insertion technique (*n* = 210).

Variables	Insertion technique		*P‐value*
	Manual (*n* = 105) *n* (%)	Forceps (*n* = 105) *n* (%)	
Age (years)			0.463
<20	24 (22.9)	25 (23.8)	
20–29	56 (53.3)	52 (49.5)	
30–39	23 (21.9)	26 (24.8)	
≥40	2 (1.9)	2 (1.9)	
Skin color			0.549
White	42 (40.0)	43 (41.0)	
Black	12 (11.4)	17 (16.2)	
Multiracial	51 (48.6)	45 (42.8)	
Number of deliveries (including the present one)			0.890
1	42 (40.0)	43 (41.0)	
2	29 (27.6)	26 (24.8)	
≥3	34 (32.4)	36 (34.2)	
Marital status			0.841
With a partner	90 (85.7)	91 (86.7)	
Without a partner	15 (14.3)	14 (13.3)	
Type of vaginal delivery			0.552
Non‐instrumental birth	100 (95.2)	98 (93.3)	
Instrumental birth	5 (4.8)	7 (6.7)	
Gestational age at birth (weeks) (mean ± SD)	38.4 ± 1.9	38.5 ± 1.1	0.543
Years of schooling			0.750
1–8	17 (16.2)	20 (19.0)	
9–12	73 (69.5)	73 (69.5)	
≥13	15 (14.3)	12 (11.5)	
Social class^a^			0.704
Upper class	0 (0)	1 (1.2)	
Upper middle class	2 (2.6)	4 (4.9)	
Lower middle class	24 (31.6)	24 (29.7)	
Poor and extremely poor	50 (65.8)	53 (64.6)	

Abbreviation: IUD, intrauterine device.

^a^ The classification of social classes in Brazil is based on the number of minimum wages (currently US$240 per month) earned per person in a household. A, upper class: above 15 minimum wages; B, upper middle class: between five and 15 minimum wages; C, lower middle class: between three and five minimum wages; D and E, poor and extremely poor: up to two minimum wages.^26^

No cases of uterine perforation, intrauterine infection, or prolonged lochia occurred following insertion or during follow‐up. The complete expulsion rate within 12 weeks postpartum was 20.5% (37/180). The “Manual” group had a complete expulsion rate of 16.7% (16/96), while the “Forceps” group presented a rate of 25% (21/84) (*P* = 0.168). Regarding the time until expulsion, most cases (64.9%) occurred within 2 weeks postpartum (minimum = 1 day; maximum = 62 days; mean = 16.8 ± 16.7 days), with no significant difference between the two groups (*P* = 0.731) (Table 2).

**TABLE 2:** Expulsion and pain during postplacental TCu380A IUD placement after vaginal delivery.

Variables	Insertion technique		Total	*P*‐value
	Manual (*n* = 96) *n* (%)	Forceps (*n* = 87) *n* (%)	(*n* = 183) *n* (%)	
Complete expulsion	16 (16.7)	21 (25)	37 (20.5)	0.168
Time until expulsion (weeks)			Total (*n* = 37)	0.731
<2	9/16 (56.2)	15/21 (71.4)	24 (64.9)	
2–6	6/16 (37.5)	5/21 (23.8)	11 (29.7)	
>6	1/16 (6.3)	1/21 (4.8)	2 (5.4)	
Pain (VAS)			Total (*n* = 160)	**0.001**
No pain (0)	27 (36)	43 (50.6)	70 (43.7)	
Mild pain (1–3)	9 (12)	19 (22.4)	28 (17.5)	
Moderate pain (4–6)	24 (32)	7 (8.2)	31 (19.4)	
Severe pain (7–10)	15 (20)	16 (18.8)	31 (19.4)	
Pain with epidural			Total (*n* = 117)	**0.002**
No pain	26 (45.6)	40 (66.7)	66 (56.4)	
Mild pain	9 (15.8)	14 (23.3)	23 (19.6)	
Moderate pain	17 (29.8)	3 (5)	20 (17.1)	
Severe pain	5 (8.8)	3 (5)	8 (6.9)	
Pain without epidural			Total (*n* = 43)	0.098
No pain	1 (5.6)	3 (12)	4 (9.3)	
Mild pain	0 (0)	5 (20)	5 (11.6)	
Moderate pain	7 (38.9)	4 (16)	11 (25.6)	
Severe pain	10 (55.6)	13 (52)	23 (53.5)	

*Note*: Bold indicates statistical significance (<0.05). In the overall analysis (without considering epidural), manual insertion was more painful (p=0.001). Even in the group of patients with epidural, manual insertion was also more painful (p=0.002).

Abbreviations: IUD, intrauterine device; VAS, visual analog scale.

Most study participants received labor analgesia (72.8%). Among the 160 women who reported their post‐insertion pain levels, 61.2% experienced no pain (VAS = 0) or mild pain (VAS between 1 and 3) during the procedure. Participants undergoing manual insertion reported higher pain levels than those with forceps insertion (*P* = 0.001). Among patients who received epidural analgesia, manual insertion remained associated with greater pain (*P* = 0.002).

Table 2 summarizes data on expulsion and pain according to the insertion technique.

Throughout the study recruitment period, there was no increased trend of IUD expulsion during the initial months following the admission of new medical residents (residency begins in March of each year) (*P* = 0.057). Further, no difference was observed when analyzing the two groups separately (Manual: *P* = 0.172; Ring Forceps: *P* = 0.169).

## 4 DISCUSSION

In this study, forceps insertion showed a higher expulsion rate; however, the difference was not statistically significant (25.0% vs. 16.7%; *P* = 0.168). Moreover, no other complications, such as uterine perforation, infection, or prolonged lochia, were observed. A study by Xu et al. (1999)^9^—the only randomized trial comparing both techniques—found similar 12‐week results in 910 Chinese women (11.3% vs. 10.8%, *P* > 0.005). Similarly, no other complications were reported, corroborating the safety of the procedure.^8^, ^9^

A recently published retrospective cohort study involving 219 women who received postplacental IUDs after vaginal delivery (117 inserted manually and 102 with ring forceps) reported a 2.5 times higher likelihood of expulsion with forceps insertion compared to manual insertion (30.4% vs. 16.2%, 95% confidence interval 1.28–4.90).^17^

Notably, vaginal delivery carries a fourfold higher expulsion risk than cesarean delivery^18^, ^19^ due to the inherent difficulty of reaching the uterine fundus. The main challenge of each insertion technique lies in overcoming the difficulty posed by the size of the puerperal uterus, which complicates access to the uterine fundus via the vaginal route. Additionally, uterine involution might displace a previously well‐positioned IUD toward the cervix.^12^

Expulsions following vaginal delivery tend to occur early,^2^, ^3^, ^8^, ^9^, ^20^ as seen in this study, with the majority (64.9%) occurring within 15 days. This situation might facilitate arranging for a reinsertion of the IUD, as participants often maintain contact with the healthcare service where they gave birth and, in this case, had postpartum follow‐up appointments scheduled between 6 and 12 weeks.

The complete expulsion rate in this study was 20.5%, consistent with results in the literature, which report expulsion rates ranging from 4.8% to 43.1% (mean 12.4%) when considering the same mode of delivery, insertion timing, and device type.^19^, ^21^

The postpartum follow‐up absenteeism rate, a time traditionally used to offer contraception, was 21.4%, despite telephone reminders and flexible scheduling. A previous study at the same service with 1629 women found an even higher absenteeism rate of 34.8%, with multiparity, psychoactive substance use, and short interbirth intervals linked to missed follow‐up visits.^22^

Most participants in this study were young, multiparous women living in poverty, a condition of socioeconomic vulnerability and a higher likelihood of unplanned pregnancies. These women face significant logistical and financial barriers to accessing reproductive planning services.^23^, ^24^

Manual insertion is technically simple and suitable for resource‐limited settings, as it does not require additional instruments beyond the IUD itself. However, because the hand is larger than the forceps, it might cause more pain during insertion,^20^ which is consistent with this study's findings. Overall, post‐vaginal delivery IUD insertion, regardless of the technique, was well tolerated, with most participants reporting “no pain or mild pain.” It is important to note, however, that pain assessment following vaginal delivery is limited due to the inherent pain associated with childbirth and the immediate postpartum period, which may hinder patients' ability to distinguish pain directly related to IUD insertion.^25^

Stable expulsion rates despite new resident admissions likely reflect continuous and repeated training. Supervision by experienced staff helped ensure technically appropriate insertions. Conducting the study in a teaching hospital with structured training and supervision likely improved outcomes. In other settings, cohesive teams and the provision of regular training might be more challenging to maintain. In contrast, the involvement of many professionals performing the procedure could be considered a potential drawback and a limitation of the study.

Despite supervision and training, residents might not yet have achieved optimal proficiency, potentially affecting results.

It is also necessary to consider the possibility that devices not fully expelled might still be located in the endocervical canal (partial expulsion) or in inverted or rotated positions. In such cases, longer follow‐up periods and ultrasound evaluations would be valuable, underscoring the ongoing challenge of ensuring patients return to healthcare services.

## 5 CONCLUSION

The postplacental IUD expulsion rate was lower with manual insertion, although not statistically significant. Expulsions occurred early and were easily identified by participants. Forceps insertion was more comfortable, although both techniques were well tolerated. The absence of analgesia, forceps, or ultrasound follow‐up should not hinder access to postplacental copper IUDs. Teaching different insertion techniques in a teaching hospital did not increase expulsion rates, and having well‐trained professionals is an important strategy for overcoming barriers to postplacental IUD provision.

## AUTHOR CONTRIBUTIONS

TBH contributed to the conceptualization of the study, project administration, methodology, investigation, formal analysis, and writing of the original draft and editing. AALB and CRTJ contributed to data curation and writing—review and editing. PMR contributed to the methodology and writing—review and editing. FGCS contributed to the conceptualization of the study, project administration, and writing—review and editing. All authors agree with the final version of the manuscript and its submission to *IJGO*.

## FUNDING INFORMATION

This study was carried out with the researchers' own funding.

## CONFLICT OF INTEREST STATEMENT

The authors have no conflicts of interest to declare.

## Supporting information


Data S1.

Data S2.

Data S3.

## Data Availability

The data that support the findings of this study are openly available in: Herculano TB, Batista AAL, Rehder PM, Surita FGdC. Data from: Randomized Controlled Trial Database on Postplacental Intrauterine Device Insertion. 2025;DRAFT VERSION. Deposited Mon Jan 20 09:26:48 BRT 2025 at http://doi.org/10.25824/redu/5KVJJY, reference number 15.
