# Supplementary material for: Manual versus forceps postplacental IUD insertion after vaginal delivery: A randomized clinical trial
Source: Int J Gynaecol Obstet. 2025 Jul 7;172(1):510–7. doi: 10.1002/ijgo.70355 (PMC12724020; doi:10.1002/ijgo.70355)

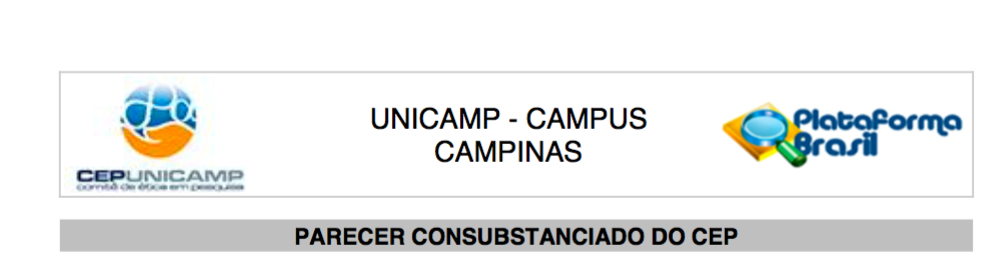


**Institutional Review Board**

**RESEARCH PROJECT DATA**

**Research Title**: COMPARISON BETWEEN TWO METHODS OF IMMEDIATE POST-PLACENTAL INSERTION OF COPPER INTRAUTERINE DEVICE IN VAGINAL BIRTH

**Researcher**: Patricia Moretti Rehder

**CAAE:** 50497321.4.0000.5404

**Proposing Institution**: Hospital da Mulher Prof. Dr. José Aristodemo Pinotti - CAISM **Main Sponsor**: Own Financing

**Number**: 4.986.281

**Comments and Considerations on the Research:**

This protocol refers to the third version of the Research Project entitled “COMPARISON BETWEEN TWO METHODS OF IMMEDIATE POST-PLACENTAL INSERTION OF COPPER INTRAUTERINE DEVICE IN VAGINAL BIRTH”, whose responsible researcher is Profa. Dr. Patricia Moretti Rehder with the collaboration of the research team formed by Thuany Bento Herculano (Master's Student, Assistant Researcher), Professor Cássia Raquel Teatin Juliato (Professor Associate Professor, Department of Tocogynecology, FCM/UNICAMP), and Profa. Dr. Fernanda Garanhani de Castro Surita (Associate Professor, Department of Tocogynecology, FCM/UNICAMP). The research falls within the "Health Sciences" area and will support the researcher's Master's Dissertation. The proposing institution is the Hospital da Mulher Prof. Dr. José Aristodemo Pinotti-CAISM from UNICAMP. According to the Basic Information of the Project, the research has an estimated budget of R$ 1200.00 and the schedule presented begins after the approval of the CEP and ends after 12 months. This is a cross-sectional study comparing two treatment groups. A total of 186 people will be approached in 2 (two) groups identified as “MANUAL INSERTION” (93 participants, INSERTION OF THE IUD MANUALLY) and “INSERTION WITH CLAMPS” (93 participants, INSERTION OF THE IUD WITH MODIFIED KELLY CLAMPS). This project aims to compare “the rate of expulsion and discomfort between manual insertion and use of Kelly Forceps after a master's change of IUD immediate delivery”.

Final Considerations at the discretion of the CEP:

- The research participant must receive a copy of the Free and Informed Consent Form, in full, signed by him (when applicable).

- Research participants are free to refuse to participate or to withdraw their consent at any stage of the research, without any penalty and without prejudice to their care (when applicable).

- The researcher must develop the research as outlined in the approved protocol. If the researcher considers discontinuation of the study, this must be justified and only performed after analysis of the reasons for discontinuation by the CEP that approved it. The researcher must wait for the CEP's opinion regarding the discontinuation, except when noticing unforeseen risk or damage to the participant or when verifying the superiority of a diagnostic or therapeutic strategy offered to one of the research groups, that is, only in case of need for immediate action to protect participants.

- The CEP must be informed of all adverse effects or relevant facts that alter the normal course of the study. It is the researcher's role to ensure adequate immediate measures in the event of a serious adverse event that has occurred (even if it has been in another center) and to send a notification to the CEP and the National Health Surveillance Agency - ANVISA - along with their position.

- Any modifications or amendments to the protocol must be presented to the CEP in a clear and succinct manner, identifying the part of the protocol to be modified and its justifications and awaiting the CEP's approval for the continuation of the research. In the case of Group I or II projects previously submitted to ANVISA, the researcher or sponsor must also send them to ANVISA, together with the CEP's approval report, to be added to the initial protocol.

- Partial and final reports must be submitted to the CEP, initially six months after the date of this approval report and at the end of the study.

-We remind you that according to Resolution 466/2012, item XI.2 letter e, “it is up to the researcher to present data requested by the CEP or CONEP at any time”.

-The researcher must keep the research data in a file, physical or digital, under their custody and responsibility, for a period of 5 years after the end of the research.

**IRB Status: Approved**


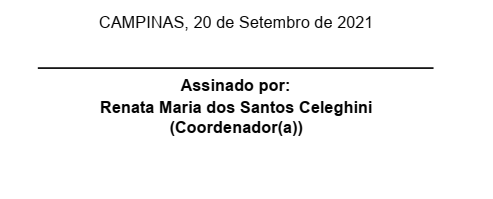


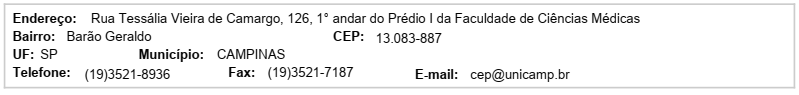

Supplement: Supplementary file 1 — Data S1. [file IJGO-172-510-s001.docx]
