# Supplementary material for: Manual versus forceps postplacental IUD insertion after vaginal delivery: A randomized clinical trial
Source: Int J Gynaecol Obstet. 2025 Jul 7;172(1):510–7. doi: 10.1002/ijgo.70355 (PMC12724020; doi:10.1002/ijgo.70355)
Supplement: Supplementary file 2 — Data S2. [file IJGO-172-510-s003.pdf]

Public trial

RBR-4j62jv6 Comparison of different ways to insert the Copper IUD in women after vaginal delivery

Date of registration: 11/12/2021 (mm/dd/yyyy)

Last approval date : 11/12/2021 (mm/dd/yyyy)

Study type:

Interventional

Scientific title:

en
Comparison of Copper Intrauterine Device Insertion Methods in the immediate postpartum

pt-br
Comparação entre métodos de Inserção do Dispositivo Intrauterino de Cobre no pós-parto imediato

es
Comparison of Copper Intrauterine Device Insertion Methods in the immediate postpartum

Trial identification

- **UTN code:**
- **Public title:**

  en
  Comparison of different ways to insert the Copper IUD in women after vaginal delivery

  pt-br
  Comparação entre diferentes formas de Inserir o DIU de Cobre em mulheres após o parto vaginal
- **Scientific acronym:**
- **Public acronym:**
- **Secondaries identifiers:**
  - **U1111-1270-0142**
    Issuing authority:
  - **50497321.4.0000.5404**
    Issuing authority: Órgão emissor: Plataforma Brasil
  - **4.986.281**
    Issuing authority: Órgão emissor: Comitê de Ética em Pesquisa da Universidade Estadual de Campinas

Sponsors

- **Primary sponsor:** Hospital da Mulher Prof. Dr. J. A. Pinotti-Caism/Unicamp
- **Secondary sponsor:**
  - **Institution:** Faculdade de Ciências Médicas da Universidade Estadual de Campinas
- **Supporting source:**

- **Institution:** Hospital da Mulher Prof. Dr. J. A. Pinotti-Caism/Unicamp

Health conditions

- **Health conditions:**

en	pt-br
Contraception; Intrauterine Devices; Postpartum Period	Anticoncepção; Dispositivos Intrauterinos; Período Pós-Parto

- **General descriptors for health conditions:**

en	pt-br
**E02.875.194** Anticoncepção	**E02.875.194** Contraception

- **Specific descriptors:**

en	pt-br
**G08.686.702** Postpartum Period	**G08.686.702** Período Pós-Parto
**E07.190.250.510** Intrauterine Devices	**E07.190.250.510** Dispositivos Intrauterinos

Interventions

- **Interventions:**

en	pt-br
Group 1: 93 women who delivered vaginally will receive a manually inserted copper IUD. Group 2: 93 women who had a vaginal delivery will receive the Copper IUD inserted with the aid of forceps. In both groups, the IUD will be inserted 10 minutes after placental delivery. To compare the expulsion rate between the groups, the 196 women will undergo revision 40 to 60 days after delivery with transvaginal ultrasound.	Grupo 1: 93 mulheres que tiveram parto vaginal receberão DIU de cobre inserido manualmente. Grupo 2: 93 mulheres que tiveram parto vaginal receberão o DIU de Cobre inserido com auxílio de uma pinça. Em ambos os grupos, o DIU será inserido 10 minutos após a saída da placenta. Para comparar a taxa de expulsão entre os grupos, as 196 mulheres passarão por revisão 40 a 60 dias após o parto com realização de ultrassonografia transvaginal.

- **Descriptors:**

en	pt-br
**E02.875.194.589** Long-Acting Reversible Contraception	**E02.875.194.589** Contracepção Reversível de Longo Prazo

Recruitment

- **Study status:** Not yet recruiting
- **Countries**
  - Brazil
- **Date first enrollment:** 12/01/2021 (mm/dd/yyyy)
-

Target sample size:	Gender:	Minimum age:	Maximum age:
186	F	18 Y	43 Y

- **Inclusion criteria:**

en

Pregnant women between 18 and 43 years old; hemoglobin greater than 8.0 mg/dl during prenatal care; single pregnancy; desire to use IUD as a contraceptive method

pt-br

Gestantes entre 18 e 43 anos; hemoglobina maior que 8,0 mg/dl durante o pré-natal; gestação única; desejo de utilizar DIU como método contraceptivo

- **Exclusion criteria:**

en

Pregnant women with diagnosis or suspicion of ovular membrane infection; diagnosis of any other infection (anywhere); active sexually transmitted infection; uterine malformation (bicornuate, septate uterus); uterine fibroids that deform the cavity; pregnant women with scheduled cesarean

pt-br

Gestantes com diagnóstico ou suspeita de infecção de membranas ovulares; diagnóstico de alguma outra infecção (em qualquer sítio); infecção sexualmente transmissível ativa; malformação uterina (útero bicorno, septado); miomatose uterina que deforme a cavidade; gestantes com cesárea agendada

Study type

- **Study design:**

en

Expanded access program	Purpose	Intervention assignment	Number of arms	Masking type	Allocation	Study phase
1	Other	Parallel	2	Open	Randomized-controlled	N/A

Outcomes

- **Primary outcomes:**

en

Lower rate of IUD expulsion in women who received the manually inserted device as assessed by transvaginal ultrasound 40 to 60 days after delivery. At least 25% fewer expulsions are expected in the manual insertion group.

pt-br

Menor taxa de expulsão do DIU em mulheres que receberam o dispositivo inserido manualmente, avaliada através de ultrassonografia transvaginal 40 a 60 dias após o parto. Espera-se, pelo menos, 25% menos expulsões no grupo com inserção manual.

- **Secondary outcomes:**

en

Level of satisfaction with the copper IUD above 70%, assessed by the Likert scale applied in the review visit 40 to 60 days after delivery, regardless of the insertion method.

pt-br

Nível de satisfação com o DIU de cobre acima de 70%, avaliado pela Escala de Likert aplicada na consulta de revisão 40 a 60 dias após o parto, independente do método para inserção.

en

Lower level of pain in patients who received the copper IUD inserted with forceps, assessed by the Visual Analog Scale with a [illegible]

pt-br

Menor nível de dor nas pacientes que receberam o DIU de cobre inserido com a pinça, avaliado pela Escala Visual Analógica [illegible]

graduation from 0 (no pain) to 10 (worst pain ever experienced). At least 2 less pain points are expected.	com graduação entre 0 (sem dor) a 10 (pior dor já experimentada). Espera-se, pelo menos, 2 pontos a menos de dor.

Contacts

- **Public contact**
  - **Full name:** Thuany Bento Herculano
  -
    - **Address:** Rua Antonio Pierozzi, n 273, Casa C, Barão Geraldo
    - **City:** Campinas / Brazil
    - **Zip code:** 13084075
  - **Phone:** 5583999044648
  - **Email:** thuany_herc@hotmail.com
  - **Affiliation:**
- **Scientific contact**
  - **Full name:** Thuany Bento Herculano
  -
    - **Address:** Rua Antonio Pierozzi, n 273, Casa C, Barão Geraldo
    - **City:** Campinas / Brazil
    - **Zip code:** 13084075
  - **Phone:** 5583999044648
  - **Email:** thuany_herc@hotmail.com
  - **Affiliation:**
- **Site contact**
  - **Full name:** Thuany Bento Herculano
  -
    - **Address:** Rua Antonio Pierozzi, n 273, Casa C, Barão Geraldo
    - **City:** Campinas / Brazil
    - **Zip code:** 13084075
  - **Phone:** 5583999044648
  - **Email:** thuany_herc@hotmail.com
  - **Affiliation:**

Additional links:

- Download in ICTRP format
