# Supplementary material for: Manual versus forceps postplacental IUD insertion after vaginal delivery: A randomized clinical trial
Source: Int J Gynaecol Obstet. 2025 Jul 7;172(1):510–7. doi: 10.1002/ijgo.70355 (PMC12724020; doi:10.1002/ijgo.70355)
Supplement: Supplementary file 3 — Data S3. [file IJGO-172-510-s002.docx]

FREE AND INFORMED CONSENT FORM

**“COMPARISON BETWEEN MANUAL OR USING FORCES INSERTION FOR COPPER INTRAUTERINE DEVICES IN THE IMMEDIATE POST-PARTUM PERIOD – RANDOMIZED CLINICAL TRIAL”**

Responsible researcher: Thuany Bento Herculano

Other researchers: Patrícia Moretti Rehder , Cássia Raquel Teatin Juliato ,​​​​

**CAAE Number: 50497321.4.0000.5404**

You are being invited to participate as a volunteer in a research study. This document, called the Informed Consent Form, aims to ensure the rights of the participant and is prepared in two copies. You and the researcher must initial all pages and sign at the end of the document. One copy should remain with you and the other with the researcher.

Please read it carefully and calmly, taking the opportunity to clarify any doubts you may have. If you have any questions before or after signing it, you can clarify them with the researcher.

**Justification and objectives:**

The objective of this study is to compare the results of manual insertion of the copper intrauterine device (IUD) in the immediate postpartum vaginal period versus the use of the modified Kelly clamp, to be used as a contraceptive method. The study will also aim to determine the acceptance and satisfaction of women regarding this contraceptive method. By participating in the study, you will contribute to the knowledge of IUD insertion in the immediate postpartum period, which has been considered a good option to increase coverage and access to contraception after childbirth. The insertion is safe, but with higher expulsion rates than if the IUD were inserted longer after childbirth. The vast majority of studies have involved manual insertion of the copper IUD. Regarding the use of the clamp, there are few studies.

**Procedures:**

By agreeing to participate in this study, data about the period in which you were pregnant and gave birth, as well as data about your postpartum period, will be collected from your prenatal card, medical records or asked by a research staff member. The copper IUD will be inserted immediately after the placenta is removed (delivered), by insertion by the obstetrician's hands or with the help of forceps. You will not be able to decide which insertion method to use.

You will need to return for a medical consultation, where the IUD will be assessed and an ultrasound will be performed. The consultation will be scheduled before you are discharged from the hospital, at your convenience and according to the availability of the service between 40 and 60 days after delivery. In addition, your satisfaction with the contraceptive method you are using will be assessed and, if you are not satisfied, another method will be offered.

If any question or procedure causes you discomfort, you may refuse to answer or perform the procedure.

The results of the research will be available when the research is completed. The data and instruments used in the research will be archived with the researcher in charge for a period of five years and after that period they will be destroyed. If you do not wish to participate in this study, there will be no prejudice to your care at CAISM.

**Discomforts and risks:**

This research involves foreseeable and inherent risks of IUD use, such as bleeding, pain, infection and uterine perforation, in addition to method failure. No method is 100% safe regarding the risk of pregnancy. Despite low pregnancy rates with IUD use, this is a possible risk. If a failure occurs, you will be referred for specialized monitoring at CAISM. You should **not** participate in this study if you are not comfortable answering the requested questions, for any reason, or if you do not feel comfortable with the subject.

**Benefits:**

By participating in the study, you will benefit from the start of contraception immediately after birth with a long-term, highly effective and low-risk contraceptive method, as well as the possibility of monitoring in the service and changing the method, according to your choice, if you so wish. In addition, it will provide improved care and knowledge about this contraceptive method.

**Alternative methods:**

There are other contraceptive methods that you can use after giving birth, such as pills, quarterly injections or subdermal progesterone implants. The IUD has the advantage of being a long-lasting method, with excellent efficacy and administration in a single time **.**

**Monitoring and assistance:**

Any questions that may arise during the research regarding IUD, clinical follow-up and other questions related to the postpartum period will be clarified. The results of the tests resulting from this research will be provided at the time of the consultation. In the case of emergencies, you should go to the CAISM emergency room and call the researchers Thuany Bento Herculano, Patrícia Moretti Rehder , Cássia Raquel Teatin Juliato , Fernanda Garanhani de Castro Surita . If you need the exams, a copy can be requested from the doctor responsible for the research.

**Confidentiality and privacy:**

Your identity will be kept confidential and no information will be given to anyone outside the research team. Your name will not be mentioned when the results of this study are published. Furthermore, the results of the study will not be included in your medical records.

**Compensation and compensation:**

The study will be conducted during your hospitalization and during your birth review appointment, and you will not need to come at another time. Your care will not be compromised or delayed. If, eventually, a visit outside of your routine is necessary, all of your expenses to participate in the study will be fully reimbursed. You will be guaranteed the right to compensation for any damages resulting from the study.

**Contact:**

If you have any questions about the study, you can contact the researchers Thuany Bento Herculano, Patrícia Moretti Rehder , Cássia Raquel Teatin Juliato , Fernanda Garanhani de Castro Surita by phone (83) 99904-4648 or at the Street: Tessália Vieira de Camargo, 126; Zip Code 13083-887 Campinas – SP.

In case of complaints or reports about your participation and ethical issues of the study, you can contact the secretariat of the Research Ethics Committee (CEP) of UNICAMP from 8:30 am to 1:30 pm and from 1:00 pm to 5:00 pm at Rua: Tessália Vieira de Camargo, 126; CEP 13083-887 Campinas – SP; telephone (19) 3521-8936; fax (19) 3521-7187; e-mail: [cep@fcm.unicamp.br](mailto:cep@fcm.unicamp.br)

**The Research Ethics Committee (CEP):**

The role of the CEP is to evaluate and monitor the ethical aspects of all research involving human beings. The National Commission for Research Ethics (CONEP) aims to develop regulations on the protection of human beings involved in research. It plays a coordinating role in the network of Research Ethics Committees ( CEPs ) of institutions, in addition to assuming the role of advisory body in the area of research ethics.

**Free and informed consent:**

After having received clarifications about the nature of the research, its objectives, methods, expected benefits, potential risks and the inconvenience that it may cause, I agree to participate and declare that I am receiving an original copy of this document initialed and signed by the researcher and myself, with all pages initialed by us:

Full Name: _____________________________________________________________

Telephone contact:____________________________________________________________

e-mail (optional):____________________________________________________________

_____________________________________________________________________

(Participant signature)

**Researcher's Responsibility:**

I hereby assure that I have complied with the requirements of resolution 466/2012 CNS/MS and complementary requirements in the preparation of the protocol and in obtaining this Free and Informed Consent Form. I also assure that I have explained and provided a copy of this document to the participant. I hereby inform that the study was approved by the CEP to which the project was presented. I undertake to use the material and data obtained in this research exclusively for the purposes provided for in this document or in accordance with the consent given by the participant.

_____________________________________________________________________

(Researcher's signature) Date: ____/_____/____
